# Passive leg raising uncovers venous congestion: dynamic fluid intolerance and the Doppler Starling curve

**DOI:** 10.1186/s13054-024-05171-z

**Published:** 2024-11-25

**Authors:** Jon-Emile S. Kenny

**Affiliations:** 1 Health Sciences North Research Institute, 56 Walford Rd, Sudbury, ON P3E 2H2 Canada; 2 Flosonics Medical, Toronto, ON Canada

## Background

Morosanu and colleagues have recently published a fascinating pilot study in *Critical Care* [1]. Following elective coronary artery bypass grafting (CABG), patients were enrolled who were mechanically-ventilated within 6 h of admission to the intensive care unit (ICU) and who had acute circulatory failure. In these patients, the authors measured the change in portal vein pulsatility index (PVPI, i.e., as a surrogate of venous filling/congestion) and the left ventricular outflow tract velocity time integral (LVOT VTI, i.e., as a surrogate of stroke volume) at 5 time points: at baseline (T_1_), one minute (T_2_) into a passive leg raise (PLR), and two minutes after returning to the semi-recumbent position (T_3_). Then, if the patient had both low PVPI and an LVOT VTI change of at least 12% during the PLR (i.e., the patient was considered *both* fluid tolerant and responsive, respectively), the patient received 7 mL/kg of Lactated Ringer’s solution (LR) over 10 min. Two additional measures were then taken: 2 min (T_4_) and 20 min (T_5_) following completion of the LR infusion. The authors examined the incidence of venous congestion (VC, i.e., defined as a PVPI ≥ 50%) following the LR and whether the antecedent PLR could predict VC. As well, they reported adverse clinical outcomes (e.g., ICU length of stay and acute kidney injury) and other echocardiographic measures as a pilot investigation.

The authors included 40 patients in their analysis with measures at T_1_-T_5_; in nearly one-half of patients, VC (i.e., PVPI of at least 50%) was observed at T_4_ (i.e., early VC), though this fell to only 5% at T_5_. Patients with early VC had significantly higher central venous pressure, worse baseline right ventricular function, and a higher incidence of severe AKI. Finally, the PVPI at T_2_ (i.e., during the PLR) predicted early VC with an area under the curve of 0.998, using a threshold of 44.3%.

## Approach to IV Fluids

When giving IV fluids, 3 basic questions should be answered: 1.) is there an indication for IV fluids? 2.) are IV fluids safe? and 3.) are IV fluids effective [2]? By enrolling only patients with signs of peripheral hypo-perfusion, the answer to the first question for the patients enrolled by Morosanu and colleagues was ‘yes.’ Safety of IV fluid can be considered within the framework of ‘tolerance’ versus ‘intolerance’ [3, 4]. By including only patients with a low PVPI as a sign of low venous pressure, we presume that the post-CABG patients in the investigation of Morosanu et al. are ‘tolerant’; so, the answer to question 2 is also ‘yes’ (barring any other signals of harm such as elevated lung water, etc.). Finally, the ‘efficacy’ of IV fluid hinges upon increased venous return (and, therefore, preload) engaging the Starling mechanism and augmenting stroke volume (SV) (i.e., there is a state of ‘fluid responsiveness’). Yet, to be truly ‘effective,’ the increased SV must also meet an arterial tree with enough vasomotor tone to enhance tissue perfusion, so called ‘circulatory effectiveness [5].’ Because Morosanu and colleagues only investigated patients with a clinically-significant increase in LVOT VTI during PLR, the prerequisite for ‘effective’ fluids was also present in their study.

However, questions 2 and 3 are troublesome because when we think about ‘safety’ and ‘efficacy’ there is conceptual confusion [4]. Can fluids be ‘safe,’ but ‘ineffective’ ? Can fluids be ‘unsafe,’ but ‘effective’? The answer is almost certainly ‘yes’ to both of these questions. A recent investigation by Munoz and colleagues supports the assertion that IV fluid ‘safety’ and ‘efficacy’ might diverge [6, 7]; Morosanu and colleagues reference the Doppler Starling curve – a framework proposed to help explain why [7, 8]. Below is an expansion of their work grounded upon the foundation of the Doppler Starling curve.

## The Doppler Starling curve

**Fig. 1:**
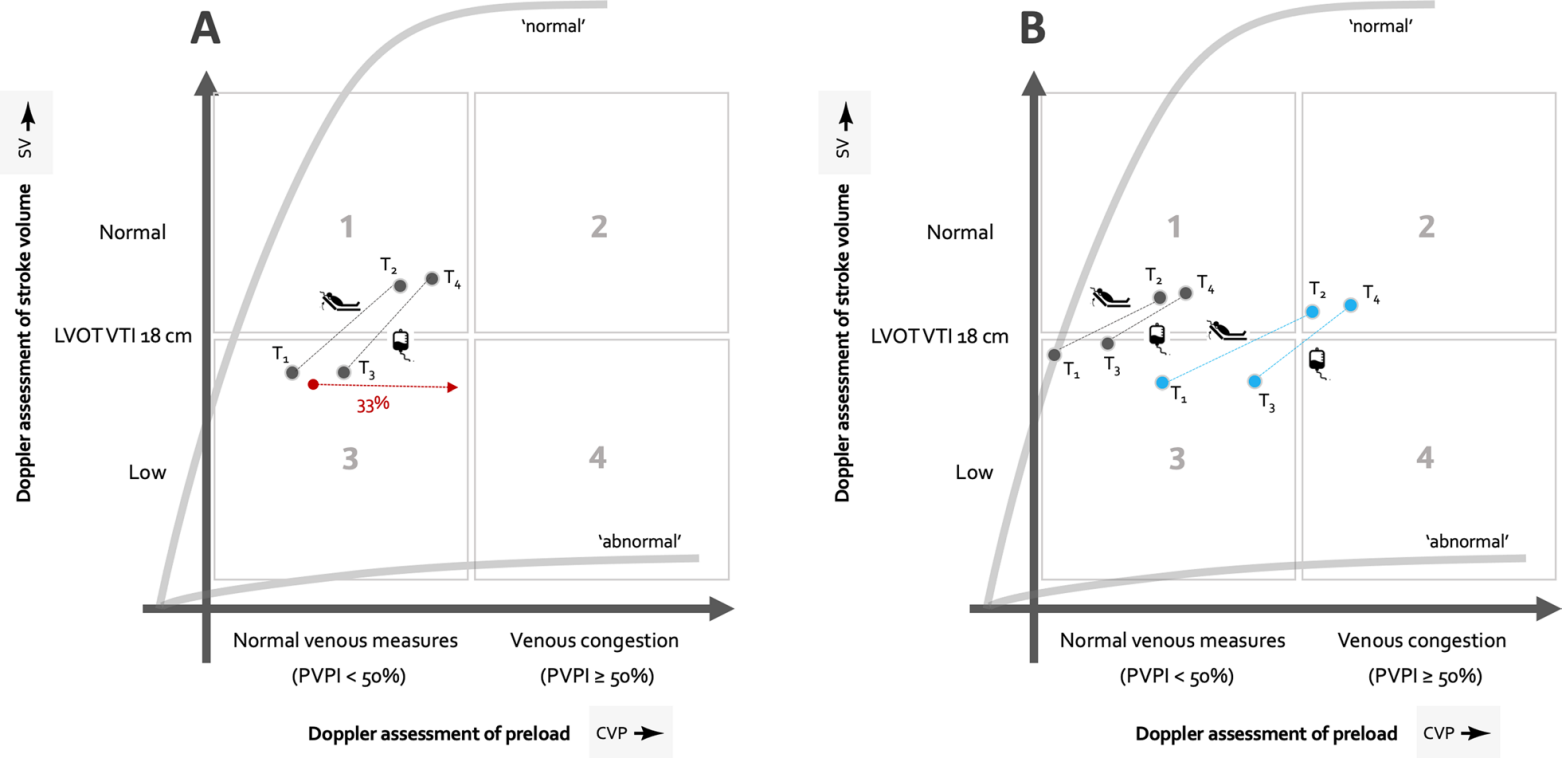
The Doppler Starling curve. The 4 hemodynamic phenotypes (1–4) are generated by combinations of normal and low stroke volume on the y-axis and normal or congested venous measures on the x-axis. A.) shows adapted data from Morosanu et.al. Fig. 2 for all patients comparing the passive leg raise (PLR) portion of the study (between T_1_ and T_2_) and the change between return to baseline and 2 min following the Lactated Ringer’s (LR) infusion (T_3_ and T_4_). T5 is excluded for clarity. The red arrow accounts for the 21 patients who were fluid tolerant but unresponsive. B.) shows the change recorded for PLR and the LR infusion when patients were split into those who did not have early congestion following LR (grey) and those who did have early congestion following LR (blue). This is adapted from Morosanu et al. Fig. 3. Based on mean values, some patients (i.e., early VC) moved from Quadrant 3 to 2 and this progression was predicted by the PLR (i.e., the change from T_1_ to T_2_). Advanced echocardiographers might argue that these patients did show signs of fluid intolerance without a PLR given their impaired, baseline right ventricular (RV) function–when the RV is no longer operating as an unstressed chamber [11]

First, Morosanu explicitly enrolled only patients with a ‘safe’ and, potentially, ‘effective’ profile as the 40 included had a decongested portal vein and were fluid responsive based upon a PLR. However, in their exclusion flow chart, there were initially 64 patients who had good echocardiographic windows and a low PVPI (i.e., fluid tolerant); of these, 21 were *fluid unresponsive*. That is to say, 33% of patients who were fluid tolerant were also fluid unresponsive. Within the Doppler Starling framework, we have previously found that 33% of patients in ‘Quadrant 3’ were fluid unresponsive [9] (see Fig. 1A below); this profile has been termed ‘dynamic fluid intolerance [4]’ because VC is expressed only with a dynamic maneuver like a PLR. Morosanu and colleagues did not record the change in PVPI in these patients but, in theory, VC would be likely. The clinical relevance of this finding is that giving fluids based only upon a baseline ‘low preload’ or ‘fluid tolerant’ profile risks giving ineffective IV fluids in a clinically significant proportion of acutely-ill patients; this is the ultrasonographic equivalent of giving fluids for a central venous pressure of less than 8 mmHg [10].

Second, Morosanu and colleagues show that patients can begin with a ‘safe’ and potentially ‘effective’ profile but, nevertheless, display another kind of ‘dynamic fluid intolerance’–moving both ‘up’ the Doppler Starling curve (i.e., on the y-axis), but also ‘out’ (i.e., along the x-axis). Based upon averages (see Fig. 1B below), these patients move from quadrant 3 to 2; the evolution of VC was predicted accurately by PLR, before IV fluids. While the slopes of the curves between those who developed early VC (blue curves) and those who did not (grey curves) were found to be statistically the same, this framework implies that with a greater range of measured values, perhaps there were subtle slope differences; this cannot be known given this pilot data. Research on the ‘slope’ of the Doppler Starling curve is underway; more specifically, whether the ratio between the LVOT VTI and VExUS + 1 (to prevent zero in the denominator) can predict patient outcome in the ICU.

In summary, Morosanu and colleagues are to be congratulated for their important pilot investigation. We should continue to anticipate divergence between venous measures and fluid responsiveness, especially with impaired cardiac function. Doppler phenotyping in this manner is an exciting avenue of active investigation.

## Data Availability

No datasets were generated or analysed during the current study.
